# A novel dinuclear iridium(III) complex as a G-quadruplex-selective probe for the luminescent switch-on detection of transcription factor HIF-1α

**DOI:** 10.1038/srep22458

**Published:** 2016-03-02

**Authors:** Lihua Lu, Modi Wang, Zhifeng Mao, Tian-Shu Kang, Xiu-Ping Chen, Jin-Jian Lu, Chung-Hang Leung, Dik-Lung Ma

**Affiliations:** 1 Department of Chemistry, Hong Kong Baptist University, Kowloon Tong, Hong Kong, China; 2 State Key Laboratory of Quality Research in Chinese Medicine, Institute of Chinese Medical Sciences, University of Macau, Macao, China

## Abstract

A novel dinuclear Ir(III) complex **5** was discovered to be specific to G-quadruplex DNA, and was utilized in a label-free G-quadruplex-based detection platform for transcription factor activity. The principle of this assay was demonstrated by using HIF-1α as a model protein. Moreover, this HIF-1α detection assay exhibited potential use for biological sample analysis.

Transcription factors are pivotal to cellular development and specialization in living organisms because of their vital role in regulating the transcription of genetic information^1^,^2^. However, the suppression or aberrant activity of transcription factors can cause various diseases, including abnormal hormone responses, developmental disorders, inflammation and cancer^3^,^4^. Consequently, the facile and sensitive detection of transcription factor activity can aid the prevention or treatment of human diseases.

Traditionally, DNA footprinting^5^,^6^, Western blotting^7^, electrophoretic mobility shift assay (EMSA)^8^, affinity chromatography^9^, and enzyme-linked immunosorbent assay (ELISA)^10^ have been used to assay transcription factor activity. However, these techniques can be costly and/or laborious for routine application^11^,^12^. Luminescence-based detection is an excellent alternative monitoring technique due to its combination of low cost, simplicity, and great sensitivity^13^,^14^,^15^,^16^,^17^. In 2011, Plaxco and co-worker designed a “transcription factor beacon” for assaying TATA binding protein, Myc-Max, or NF-kappaB (NF-κB)^18^. Moreover, an ultrasensitive assay for transcription factor detection using a helicase-dependent amplification mechanism was reported in 2013^19^. Recently, a self-assembled triplex DNA molecular switch stabilized by Ag^+^ was explored as a probe for NF-κB detection, and this assay was applied for real biological sample analysis^20^. However, those reported luminescent transcription factor detection assays typically require either amplification steps, labeled oligonucleotides, and/or multiple DNA probes.

Transition metal complexes possess several advantages for sensing applications, such as simple syntheses, tunable photophysical properties, large Stokes shifts and long phosphorescent lifetimes^21^,^22^,^23^,^24^,^25^. In 2011, our group developed the first metal-based label-free switch-on detection platform for NF-κB by using the luminescent ‘light switch’ complex [Ru(phen)_2_(dppz)]^2+^ (where phen = 1,10-phenanthroline; dppz = dihydro[3,2-*α*:2′, 3′-*c*]phenazine)^26^. More recently, our group has explored a variety of mononuclear iridium(III) complexes as G-quadruplex-selective probes for the construction of a range of label-free luminescent detection platforms^27^,^28^,^29^,^30^,^31^,^32^,^33^. The G-quadruplex structure is a well-known typical DNA secondary structure formed by planar stacks of four guanines stabilized by Hoogsteen hydrogen bonding and monovalent cations, which has been extensively used as signal transducers for the construction of label-free detection platforms^34^,^35^,^36^,^37^. We found that these mononuclear iridium(III) complexes potentially interacted with the loop regions of the G-quadruplex^27^,^28^,^29^,^30^,^31^,^32^,^33^. In this study, we sought to link two simple Ir(III) complexes without strong planarity and hydrophobicity, and then strengthen the electrostatic interaction between the complex and G-quadruplex DNA. We anticipate that the enhanced electrostatic interaction could provide a driving force for the dinuclear complex to bind to the G-quadruplex DNA. On the other hand, previous study reported that G-quadruplex binder linked with a positively-charged alkylamine through a four-carbon chain could enhance the G-quadruplex stabilizing ability, whereas too short linker will decrease the stabilizing ability^38^,^39^,^40^. Therefore, in this project we choose a linker with similar length for the design of dinuclear complex.

Inspired by these concepts, we were interested to see if we could develop a luminescent dinuclear iridium(III) complex for the G-quadruplex-based detection of the mammalian transcription factor hypoxia- inducible factor-1α (HIF-1α). HIF-1α is activated under conditions of reduced oxygen tension, and binds to hypoxia response elements containing the consensus sequence 5′-A(G)CGTG-3′^41^,^42^. HIF-1α levels have been correlated with vascularization, tumor growth and metastasis both in animal models and in clinical studies^43^,^44^,^45^. Hence, the detection of HIF-1α activity is important for cancer diagnosis and prognosis.

## Results

**Figure 1:**
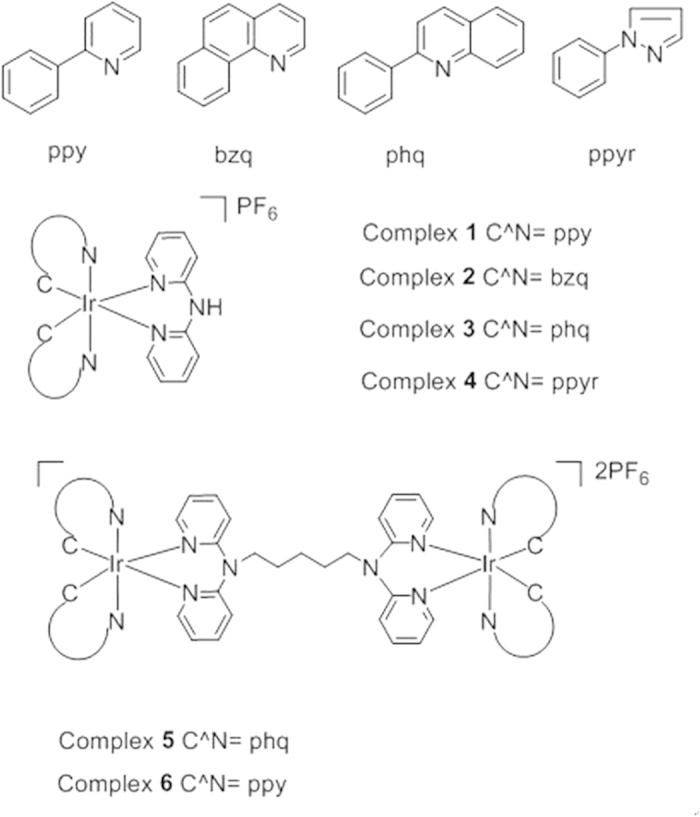
Structures of the mononuclear Ir(III) and dinuclear Ir(III) complexes used for G-quadruplex-selective probe screening.

**Figure 2:**
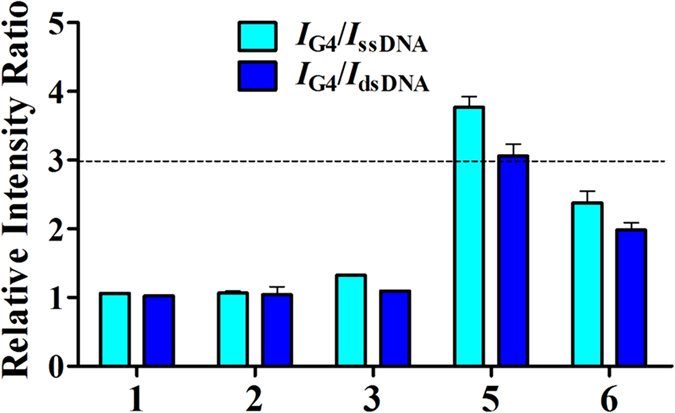
Luminescence enhancement selectivity ratios of complexes **1**–**6** for G-quadruplex DNA (PS2.M) over dsDNA (ds17) and ssDNA (CCR5-DEL). Error bars represent the standard deviations of the results from three independent experiments.

In this work, four mononuclear Ir(III) complexes **1**–**4** and two dinuclear Ir(III) complexes **5**–**6** (Fig. 1) were investigated for their ability to monitor different structures of DNA (Table S1) by emission titration. The mononuclear complexes **1**–**4** share a common di(pyridin-2-yl)amine N^N ligand, but bear different C^N ligands. The connection of the mononuclear complex **3** and **1** via a 5-carbon linker gives rise to the dinuclear metal complexes **5** and **6**. The results of the emission titration experiment showed that the nature of the C^N ligands as well as the size of the complex affected the selectivity and photophysical properties of the complexes. For instance, complex **4** containing the 1-phenyl-1H-pyrazole (ppyr) C^N ligand showed no emission under all conditions (Fig. S1). Excitingly, the dinuclear complex **5** [Ir_2_(tpptda)(phq)_4_]^2+^ (where tpptda = *N*^1^, *N*^1^, *N*^5^, *N*^5^-tetra(pyridin-2-yl)pentane-1,5-diaminee, phq = 2-phenylquinoline) showed a significant discrimination for G-quadruplex DNA over single-stranded DNA (ssDNA) and double-stranded (dsDNA), with the highest *I*_G4_/*I*_dsDNA_ and *I*_G4_/*I*_ssDNA_ ratios among the six complexes screened (Fig. 2). However, the dinuclear complex **6** does not display a strong lumincence enhancement towards PS2.M G-quadruplex. This indicates that the structure of the dinuclear Ir(III) complex plays an important role for the G-quadruplex recognition. The dinuclear complex **5** is therefore a special case in that an appropriate combination of C^N donor, N^N donor and linker length are joined together to create an effective G-quadruplex probe. Complex **5** also showed the highest absolute luminescence response towards the PS2.M (5′-GTG_3_TAG_3_CG_3_T_2_G_2_-3′) G-quadruplex (*ca*. 4.1-fold) (Fig. S1). Meanwhile, complexes **1**–**4** exhibited little or no selectivity for G-quadruplex DNA.

The synthesis and preparation of the dinuclear Ir(III) complexes **5** and **6** is presented in the Supporting Information (Scheme 1), while photophysical properties and spectroscopic characterization of complexes **1**–**6** are shown in Table S2 and Fig. S2. The absorption spectra of the complexes and their ligands show that the photophysical properties of the ligands are significantly changed upon complexation. The G-quadruplex fluorescent intercalator displacement (G4-FID) assay was used to examine the selectivity and affinity of **5** for the G-quadruplex structure^46^. Complex **5** displaced thiazole orange (TO) from the PS2.M G-quadruplex DNA with a ^G4^DC_50_ value of 7.0 μM (^G4^DC_50_ is the half-maximal concentration of compound required to displace 50% TO from DNA), which was lower than the concentration required for duplex DNA (Fig. S3a). The G- quadruplex-selectivity of complex **5** was further validated using a fluorescence resonance energy transfer (FRET)-melting assay. The melting temperature (Δ*T*_m_) of the labeled PS2.M (5′-*FAM*-GTG_3_TAG_3_CG_3_T_2_G_2_-*TAMRA*-3′) G-quadruplex was enhanced by approximately 12 °C by 3 μM of **5** (Fig. S3b). However, no significant variation in the melting temperature of F10T (5′-*FAM*-TATAGCTA-HEG-TATAGCTATAT-*TAMRA*-3′) dsDNA was found at the same concentration of **5** (Fig. S3c).

Moreover, the ability of **5** to stabilize the PS2.M G-quadruplex was not significantly changed by the addition of 10-fold excess of unlabeled ssDNA or dsDNA (ds26) competitor DNA (Fig. S3d). These results collectively show that the dinuclear Ir(III) complex **5** selectively binds to G-quadruplex DNA over ssDNA or dsDNA. To our knowledge, complex **5** is the first dinuclear Ir(III) G-quadruplex-selective probe reported in the literature.

Our previous studies have indicated that the G-quadruplex loop size may mediate the interaction between G-quadruplex structures and mononuclear Ir(III) complexes. Therefore, we explored the effect of PS2.M G-quadruplex loop size on the emission of the dinuclear Ir(III) complex **5**. The luminescence of **5** was tested in the presence of various G-quadruplexes, including sequences containing a 5′-side loop, a central loop or a 3′-side loop, with different loop sizes were tested (Fig. S4a–c, the specific sequences used in this experiments presented in Table S3). Generally, the emission intensity of complex **5** correlated positively with loop size, irrespective of the site of the loops. We also investigated the loop size effect using the PS2.M G-quadruplex sequence with different loop length (Fig. S4). As the loop length increased from 1 to 17 nt, the emission fold change of **5** increased from 4.2 to 9.2-fold for the 5′-side loop, from 4.4 to 8.7-fold for the central loop and from 4.2 to 8.4-fold for the 3′-side loop. The signal enhancement reached a maximum at a loop size of 9–13 bases for all these three loops. This result shows that the loop size affects the interaction between the dinuclear Ir(III) complex **5** and G-quadruplex DNA. We anticipate that the increase of G-quadruplex loop size could generate additional binding surface for interactoin with complex **5**. The folded loop with a negatively-charged sugar phosphate backbone may act as a binding pocket-like receptor to attact the positively-charged complex **5**.

**Figure 3:**
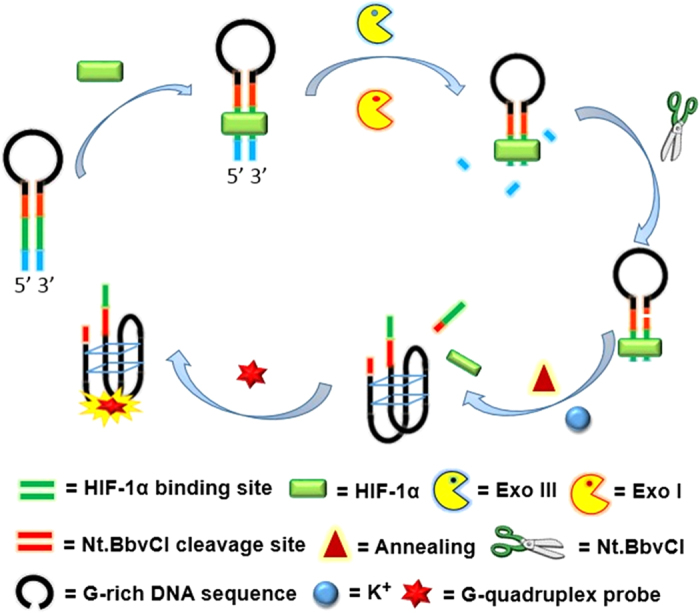
Schematic representation of the luminescent HIF-1α detection assay using a G-quadruplex probe.

Considering the promising luminescent behavior possessed by the dinuclear Ir(III) complex **5**, we sought to establish a luminescent detection platform for transcription factor activity by employing the G-quadruplex-selective **5** as a signal transducer and HIF-1α as a model protein. The principle behind our transcription factor detection platform is based on the enzymatic activity of Exo III, Exo I and nicking endonuclease Nt.BbvCI, and the G-quadruplex-selectivity of complex **5**. A hairpin DNA structure contains the HIF-1α recognition site (green line in Fig. 3) and the Nt.BbvCI cleavage site (red line in Fig. 3) in its duplex region, and a G-quadruplex-forming sequence (black line in Fig. 3) in its loop region. The addition of HIF-1α results in the formation of a HIF-1α-hairpin DNA complex due to the specific binding between HIF-1α with its cognate sequence. The HIF-1α-bound hairpin DNA structure is protected from digestion of Exo III and Exo I, thereby preserving the Nt.BbvCI cleavage site. Cleaved of the hairpin DNA by Nt.BbvCI then releases the G-quadruplex-forming fragment, which folds into a G-quadruplex structure. The nascent G-quadruplex structure is subsequently bound by the G-quadruplex-selective dinuclear Ir(III) complex **5** with an increased emission enhancement, which allows the system to act as a switch-on luminescent platform for HIF-1α detection. The addition of Exo III and Exo I ensures that the background signal of this system is low, as the hairpin DNA will be completely digested by Exo III and Exo I in the absence of HIF-1α, leading to an enhanced detection sensitivity.

Since complex **5** displays high luminescence response towards the PS2.M G-quadruplex, and only weak luminescence towards dsDNA and ssDNA, we employed PS2.M in the sensing platform. Moreover, PS2.M contains only 18 bases, allowing for a relatively short complementary sequence to be used to prevent the pre-formation of the PS2.M G-quadruplex, which could reduce the risk of the stability of dsDNA adversely influencing the sensitivity. We initially designed two hairpin DNA sequences HP1 (5′-G_2_TACGTG_2_CTGAG_2_C_2_AGTG_3_TAG_3_CG_3_T_2_G_2_C_2_TCAGC_2_ACGTAC_2_-3′) and HP2 (5′-G_2_TACGTG_2_CTGAG_2_C_2_AACGTG_3_TAG_3_CG_3_T_2_G_2_C_2_TCAGC_2_ACGTAC_2_-3′) (Fig. S5a,b). The hairpin DNA sequences were incubated, in turn, with HIF-1α, Exo III/Exo I, Nt.BbvCI and K^+^ ions. We observed that the system with HP1 exhibited a higher signal increase in response to HIF-1α compared with HP2 (Fig. S6). We reason that this is because HP3 (5′-ACGTG_2_CTGAG_2_C_2_AGTG_3_TAG_3_CG_3_T_2_G_2_C_2_-3′) (Fig. S5c), formed from the cleavage of HP1, can more easily fold into a G-quadruplex structure compared to HP4 (5′-ACGTG_2_CTGAG_2_C_2_A_2_CGTG_3_TAG_3_CG_3_T_2_G_2_C_2_-3′) (Fig. S5d), the sequence that is released from the cleavage of HP2. To confirm that the observed emission enhancement was associated with the formation of the G-quadruplex structure induced by HIF-1α, several control experiments were carried out. We incubated complex **5** with HIF-1α in the absence of hairpin DNA or enzymes. Up to 120 nM of HIF-1α induced minor change in the emission of **5**, indicating that **5** did not directly interact with HIF-1α (Fig. S7a). We then assessed whether or not the addition of Exo III and Exo I was able to reduce the background emission of the assay. The emission of complex **5** is only slightly enhanced by 1.1-fold upon the addition of HP1 due to the weak interaction of **5** with dsDNA. Digestion of the hairpin DNA by Exo III and Exo I produces short ssDNA fragments that interact very weakly with complex **5**, reducing the emission intensity to the original state (Fig. S7b). We also designed two mutant hairpin sequences to verify the mechanism of the detection assay. The first mutant HP1-M1 (5′-G_2_TA*ATAC*GCTGAG_2_C_2_AGTG_3_TAG_3_CG_3_T_2_G_2_C_2_TCAGC*GTAT*TAC_2_-3′) contains four point mutations in the HIF-1α binding site, and thus is not effectively recognized by HIF-1α. The second mutant HP1-M2 (5′-G_2_TACGTG_2_CTGAG_2_C_2_AGT*A*_3_TA*A*_3_CG_3_T_2_G_2_C_2_TCAGC_2_ACGTAC_2_-3′) contains six mutations in the G-rich tract, and therefore cannot form into a G-quadruplex structure. As expected, only a minor enhancement in the emission of **5** was found upon addition of 80 nM of HIF-1α to HP1-M1 or HP1-M2 (Fig. S8). The hairpin-to-quadruplex configurative transition caused by HIF-1α was further confirmed by circular dichroism (CD) spectroscopy (Fig. S9). In the absence of HIF-1α and enzymes, the CD spectrum of the hairpin DNA exhibits a strong positive signal at around 280 nm and a negative signal at 245 nm, which are typical CD peaks for hairpin DNA. In the addition of HIF-1α and Exo I, Exo III and Nt.BbvCI enzymes, the spectrum varies to reveal a positive signal at about 260 nm, and a weak negative signal at about 240 nm, which are typical peaks of parallel G-quadruplex^47^,^48^. Based on these results, we presume that the luminescence enhancement of the system arises from the specific binding of **5** with G-quadruplex DNA.

**Figure 4:**
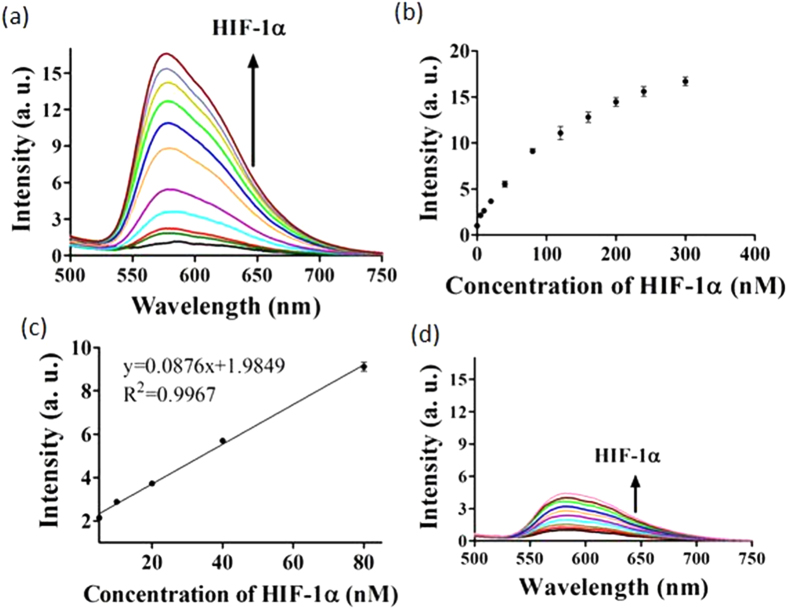
(**a**) Luminescence response of **5** to various concentrations of HIF-1α: 0, 5, 10, 20, 40, 80, 120, 160, 200, 240 and 300 nM in buffered solution. (**b**) The relationship of signal intensity (λ_Em_ = 580 nm) and the concentration of HIF-1α. (**c**) Linear plot of the signal change in luminescence intensity (λ_Em_ = 580 nm) *vs*. HIF-1α concentration. Error bars represent the standard deviations (SD) of the results from three independent experiments. (**d**) Luminescence spectra of **5** in response to various concentrations of HIF-1α: 0, 5, 10, 20, 40, 80, 120, 160, 200, 240 and 300 nM in a detection system without Nt.BbvCI.

After verification of the detection mechanism, we studied the emission response of the assay to a range of concentrations of HIF-1α. The detection platform showed a *ca*. 16.6-fold signal increase in the presence of 300 nM of HIF-1α (Fig. 4a) with a detection linear range for HIF-1α from 5 to 80 nM (Fig. 4c). The detection limit of this assay for HIF-1α was estimated to be 5 nM at a signal-to-noise ratio (S/N) of 3 (Fig. S10). This detection limit is comparable to those reported luminescence-based detection platforms for other transcription factors assay (Table S4)^15^,^49^. A further experiment was carried out to evaluate the role of the nicking endonuclease step for the detection process performing the assay without Nt.BbvCI is anticipated to generate the hairpin DNA HP5 (5′-ACGTG_2_CTGAG_2_C_2_AGTG_3_TAG_3_CG_3_T_2_G_2_C_2_TCAGC_2_ACGT-3′) (Fig. S5e), which is formed when the original hairpin DNA HP1 is partially digested by the two exonucleases up to the HIF-1α recognition site. Since HP5 is unable to fold into a G-quadruplex motif, the luminescent enhancement for this system was only about one-fourth of that in the presence of Nt.BbvCI (Fig. 4d), due to the weaker binding between complex **5** and the hairpin DNA compared with G-quadruplex DNA. This experiment demonstrates that the nicking endonuclease Nt.BbvCI is a requisite enzyme for this detection platform.

**Figure 5:**
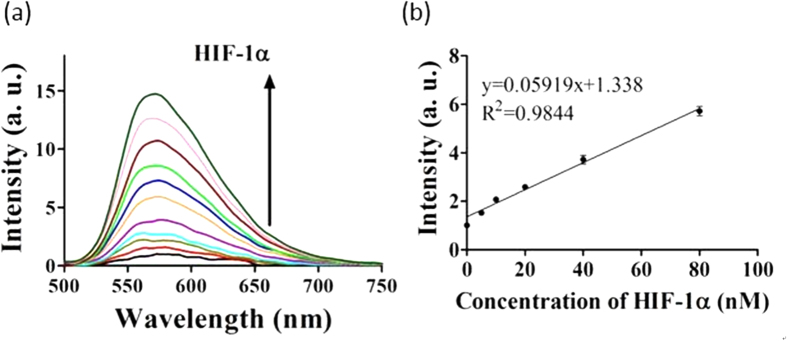
(**a**) Luminescence responses of **5** to various concentrations of HIF-1α: 0, 5, 10, 20, 40, 80, 120, 160, 200, 240 and 300 nM in diluted cell extract solution. (**b**) Linear relation of the change in luminescence intensity (λ_Em_ = 580 nm) *vs*. HIF-1α concentration. Error bars show the standard deviations of the results from three independent experiments.

The selectivity of this detection platform for HIF-1α was further examined by monitoring the signal enhancement of complex **5** to other G-quadruplex binding protein such as thrombin and insulin. As shown in Fig. S11, only weak luminescence enhancement was observed upon the addition of 0.8 μM of thrombin or insulin, and those concentrations of thrombin and insulin also did not interfere with the detection of 80 nM of HIF-1α. This result indicates that the detection platform is specific to HIF-1α and can be potentially used to detect the cellular content level of HIF-1α. We next evaluated the effectiveness of this detection platform for HIF-1α activity in diluted cellular extract. In the presence of 0.5% (*v*/*v*) cell extract, the **5**/hairpin DNA system increased in emission intensity as HIF-1α levels increased (Fig. 5a,b). This result suggests that this assay could be further optimized to detect HIF-1α levels in biological samples.

## Discussion

In conclusion, complex **5** was developed as the first dinuclear luminescent Ir(III) G-quadruplex-selective probe reported in the literature. Although the Ir(III) complex contains a positive charge that may be beneficial for G-quadruplex binding, the properties of the C^N and N^N donor ligands (such as planarity or hydrophobicity) of the Ir(III) complex also play an important role in G-quadruplex probe development. In this study, we sought to link two simple Ir(III) complexes without the classic G-quadruplex probe property such as strong planarity and hydrophobicity, and then strengthen the electrostatic interaction between the complex and G-quadruplex DNA. We anticipate that the enhanced electrostatic interaction could provide a driving force for the dinuclear complex to bind to the G-quadruplex DNA. The five-carbon chain allows complex **5** adopt an optimum conformation for selective binding with the G-quadruplex structure. Meanwhile, the selective binding of complex **5** for G-quadruplex DNA over dsDNA may be due to the bulky nature of the dinuclear complex, which prevents intercalation with dsDNA.

As the consequence, complex **5** was identified from screening a library of mononuclear and dinuclear Ir(III) complexes, and was subsequently employed for developing a label-free G-quadruplex-based assay for HIF-1α assay. The detection for HIF-1α is based on the high specificity of **5** towards G-quadruplex DNA over random coil DNA and duplex. In the presence of HIF-1α, the binding of the transcription factor to its cognate sequence protects the duplex substrate from exonuclease digestion, allowing a G-quadruplex-forming motif to be released after nicking by Nt.BbvCI. Furthermore, we demonstrated that HIF-1α levels in cell extract could be monitored using this assay. This label-free G-quadruplex-based luminescence switch-on platform is easy-to-use, user-friendly and cost-effective compared to conventional DNA footprinting, Western blotting, affinity chromatography, ELISA, and gel-based assays, which usually require complicated operation procedures and time-consuming preparation.

## Methods

### Chemicals and materials

Thrombin, insulin, and other reagents, unless specified, were purchased from Sigma Aldrich (St. Louis, MO). Iridium chloride hydrate (IrCl_3_.xH_2_O) was purchased from Precious Metals Online (Australia). Exonucease I (ExoI), Exonucease III (ExoIII) and Nb.BbvCI were purchased from New England Biolabs Inc. (Beverly, MA, USA). All oligonucleotides were synthesized by Techdragon Inc. (Hong Kong, China). The protein HIF-1α is purchased from Sino Biological Inc. (Beijing, China). The DNA secondary structures are simulated by software DNAMAN 6.0.3.99 (Lynnon Biosoft, America).

### FRET melting assay

The ability of **5** to stabilize PS2.M G-quadruplex or dsDNA was investigated using a fluorescence resonance energy transfer (FRET) melting assay. The experimental procedure was similar to previously described^50^.

### G-quadruplex fluorescent intercalator displacement (G4-FID) assay

The G4-FID experiment was used to evaluate the binding affinity of **5** to G-quadruplex DNA or dsDNA. The experiment procedure was the same as previously reported^51^.

### Detection of HIF-1α

The hairpin DNA HP1 (50 μM) in Tris buffer (10 mM, pH 7.4, 100 mM NaCl, 1 mM EDTA, final concentration) was heated to 92 °C for 10 min, cooled to room temperature at 0.1 °C/s, and further stabilized at room temperature for 1 h to ensure formation of the hairpin substrate. The annealed product was stored at –20 °C before use. For assaying of HIF-1α activity, 25 μL of transcription factor binding buffer (10 mM Tris, pH 7.4, 1 mM MgCl_2_, 50 mM KCl, 1 mM DTT, 10% glycerol), containing 2 μM HP1 DNA were incubated with the appropriate amount of HIF-1α for 30 min. Then, 25 μL 2 × NEBuffer 1 (10 mM Bis-Tris-Propane-HCl, 10 mM MgCl_2_, 1 mM DTT, pH 7.0, 25 °C), 50 units of Exo III and 40 units Exo I were added to the solution containing the hairpin DNA (1 μM,) and HIF-1α. The mixture was incubated at 37 °C for 60 min to allow digestion of non-protected hairpin DNA. The digestion reaction solution was terminated by heating at 92 °C for 10 min. This step inactivates both the exonucleases inactive and HIF-1α. The resulted mixture was cooled to 37 °C, 5 μL 10 × Cutsmart Buffer (20 mM Tris, 50 mM KCl, pH 7.2) and 25 units of Nt. BbvCI were subsequently added, and the mixture was incubated at 37 °C for 2 h. The reacted mixture was diluted with 450 μL Tris buffer (10 mM, pH 7.4) containing 100 mM K^+^. Finally, 1 μM of complex **5** was added to the mixture. Emission spectra were recorded in the 500–750 nm range using an excitation wavelength of 310 nm.

For the detection of HIF-1α activity in cell extract 20 μL of transcription factor binding buffer (10 mM Tris, pH 7.4, 1 mM MgCl_2_, 50 mM KCl, 1 mM DTT, 10% glycerol), containing 2 μM HP1 DNA was added to 5 μL cell extract, and was incubated with the appropriate amount of HIF-1α protein for 30 min. HIF-1α detection was performed as above.

### Syntheses

Complexes **1**–**4** were prepared according to (modified) literature methods^52^. All complexes were characterized by ^1^H-NMR, ^13^C-NMR, high resolution mass spectrometry (HRMS) and elemental analysis. For complex **5**, its N^N ligand, *N*-pentyl-*N*-(pyridin-2-yl)pyridin-2-amine was obtained by the following procedure. To a solution of 0.26 g (1.5 mmol) 2,2′-dipyridylamine in 5 mL anhydrous THF, 0.046 g (2.0 mmol) NaH was added under an ice bath. The mixture was stirred for 30 min, and 0.225 g (1.5 mmol) 1-bromohexane was added at room temperature. The reaction was allowed to heat reflux and monitored by TLC. After quenching the reaction by water, the mixture was diluted with EtOAc (50 mL), washed with H_2_O (2 × 15 mL) and brine (2 × 15 mL). The organic layer was dried by anhydrous Na_2_SO_4,_ and the solvent was removed under reduced pressure to get the crude product which was purified by silica gel column (PE:EA = 10:1~2:1) to yield the ligand (0.22 g, 60.9%) as a white solid.

The dinuclear Ir(III) complexes **5** and **6** were synthesized according to Scheme S1. The precursor iridium(III) complex dimer [Ir_2_(C^N)_4_Cl_2_] was prepared as reported^52^. The bridged N^N ligand, *N*^1^, *N*^1^, *N*^5^, *N*^5^-tetra(pyridin-2-yl)pentane-1,5-diaminee (tpptda) was synthesized by the following procedure. To a mixture of 2.60 g potassium hydroxide in 15 mL DMSO, 1.71 g (0.01 mol) 2,2′-dipyridylamine was added. The mixture was stirred at room temperature for 4 h and 1.62 g (0.005 mol) 1, 5-diiodopentane was added subsequently. The reaction was monitored by TLC. After quenching the reaction by 20 mL water, the solution was extracted by diethylether (4 × 30 mL). The combined organic phase was dried by anhydrous Na_2_SO_4_, the solvent was removed under reduced pressure to get the crude product which was purified by silica gel column (PE:EA = 10:1~1:1) to yield ligand B (1.40 g, 68.5%) as a yellow solid. Then, a suspension of [Ir_2_(C^N)_4_Cl_2_] (0.2 mM) and corresponding N^N ligands, tpptda), (0.24 mmol) in a mixture of DCM:CH_3_OH (1:1.1, 40 mL) was refluxed 6 h under the protection of nitrogen. The resulting solution was then cooled to room temperature, and filtered to remove unreacted dichloride-bridged Ir(III) dimer. To the filtrate, a solution of ammonium hexafluorophosphate (NH_4_PF_6_) was added and the filtrate was reduced in volume by rotary evaporation until precipitation of the crude product appeared. The precipitate was then filtered and washed with several portions of water (3 × 25 mL) followed by diethyl ether (3 × 25 mL). The mixture was purified by silica gel column dichloromethane as the eluent to yield the titled compound.

Complexes **1**–**4**. Reported^48^.

Complex **5**. Red powder, yield: 59.7%. ^1^H NMR (400 MHz, Acetone-*d*_6_) δ 8.59 (d, *J* = 8.8 Hz, 2H), 8.54 (d, *J* = 8.8 Hz, 2H), 8.48–8.44 (m, 4H), 8.20–8.14 (m, 4H), 8.08–8.03 (m, 8H), 7.69–7.61 (m, 8H), 7.57 (t, *J* = 7.6 Hz, 2H), 7.40 (t, *J* = 7.6 Hz, 2H), 7.15–6.99 (m, 16H), 6.80 (t, *J* = 7.6 Hz, 4H), 6.35 (d, *J* = 7.6 Hz, 4H), 4.13–4.05 (m, 1H), 3.88–3.81 (m, 1H), 2.74–2.67 (m, 1H), 2.60–2.53 (m, 1H), 0.86–0.60 (m, 3H), 0.59–0.48 (m, 1H), −0.67~−0.69 (m, 1H), −0.97~−1.02 (m, 1H); ^13^C NMR (100 MHz, Acetone-*d*_6_) 172.4, 157.0, 152.5, 152.4, 152.3, 149.1, 147.4, 147.3, 141.9, 141.1, 141.0, 133.8, 131.5, 130.4, 130.3, 129.2, 128.3, 127.7, 127.5, 125.8, 123.6, 122.8, 119.4, 119.3, 119.1, 52.7, 52.5, 27.0, 26.1, 25.3; MALDI-TOF-HRMS: Calcd. for C_85_H_66_Ir_2_N_10_[M–2PF_6_]^2+^: 1612.4739 Found: 601.4461 + 1011.3397 = 1612.7858; Anal.: (C_85_H_66_Ir_2_N_10_P_2_F_12_) C, H, N: calcd. 53.68, 3.50, 7.36; found. 53.86, 3.80, 6.95.

Complex **6**. Yellow powder, yield: 70.3%. ^1^H NMR (400 MHz, Acetonitrile-*d*_3_) δ 8.02–7.99 (m, 4H), 7.97–7.94 (m, 4H), 7.90–7.85 (m, 4H), 7.83–7.75 (m, 4H), 7.71–7.66 (m, 8H), 7.38 (d, *J* = 8.4 Hz, 4H), 7.06–7.00 (m, 4H), 6.92–6.87 (m, 8H), 6.77 (t, *J* = 7.6 Hz, 4H), 6.10–6.07 (m, 4H), 4.03–3.94 (m, 2H), 3.58–3.43 (m, 2H), 1.19–1.12 (m, 4H), 0.53–0.42 (m, 2H); ^13^C NMR (100 MHz, Acetonitrile-*d*_3_) 167.5, 167.4, 155.9, 150.2, 149.9, 149.6, 144.0, 139.9, 138.1, 138.0, 131.3, 129.8, 124.5, 122.6, 122.2, 121.3, 119.7, 118.1, 51.1, 51.0, 26.1, 25.9, 23.8; MALDI-TOF-HRMS: Calcd. for C_69_H_58_Ir_2_N_10_PF_6_[M–PF_6_]^+^: 1556.3746 Found: 1556.1052; Anal.: (C_69_H_58_Ir_2_N_10_P_2_F_12_) C, H, N: calcd. 48.70, 3.44, 8.23; found. 48.41, 3.25, 8.18.

## Additional Information

**How to cite this article**: Lu, L. *et al*. A novel dinuclear iridium(III) complex as a G-quadruplex-selective probe for the luminescent switch-on detection of transcription factor HIF-1α. *Sci. Rep*. **6**, 22458; doi: 10.1038/srep22458 (2016).

## Supplementary Material

Supplementary Information
