# Supplementary material for: A novel dinuclear iridium(III) complex as a G-quadruplex-selective probe for the luminescent switch-on detection of transcription factor HIF-1α
Source: Sci Rep. 2016 Mar 2;6:22458. doi: 10.1038/srep22458 (PMC4773817; doi:10.1038/srep22458)
Supplement: Supplementary Information [file srep22458-s1.doc]

**Electronic Supporting Information**

**A novel dinuclear iridium(III) complex as a G-quadruplex-selective probe for the luminescent switch-on detection of transcription factor HIF-1α**

Lihua Lu^1,§^, Modi Wang^1,§^, Zhifeng Mao^1^, Tian-Shu Kang^2^, Xiu-Ping Chen^2^, Jin-Jian Lu^2^, Chung-Hang Leung^2^ and Dik-Lung Ma*^,1^

^1^ Department of Chemistry, Hong Kong Baptist University, Kowloon Tong, Hong Kong, China E-mail: edmondma@hkbu.edu.hk

^2^ State Key Laboratory of Quality Research in Chinese Medicine, Institute of Chinese Medical Sciences, University of Macau, Macau, China

**Photophysical measurement**. Emission spectra and lifetime measurements for complexes were performed on a PTI TimeMaster C720 Spectrometer (Nitrogen laser: pulse output 337 nm) fitted with a 380 nm filter. Error limits were estimated: λ (±1 nm); τ (±10%); φ (±10%). All solvents used for the lifetime measurements were degassed using three cycles of freeze-vac-thaw.[^1^](#_ENREF_1)

Luminescence quantum yields were determined using the method of Demas and Crosby [Ru(bpy)_3_][PF_6_]_2_ in degassed acetonitrile as a standard reference solution (Φ_r_ = 0.062) and calculated according to the following equation:

Φ_s_ = Φ_r_(*B*_r_/*B*_s_)(*n*_s_/*n*_r_)^2^(*D*_s_/*D*_r_)

where the subscripts s and r refer to sample and reference standard solution respectively, *n* is the refractive index of the solvents, *D* is the integrated intensity, and Φ is the luminescence quantum yield. The quantity *B* was calculated by *B* = 1 – 10^–^*^AL^*, where *A* is the absorbance at the excitation wavelength and *L* is the optical path length.

**Total cell extract preparation**. The TRAMPC1 (ATCC® CRL2730™) cell line were purchased from American Type Culture Collection (Manassas, VA 20108 USA). Prostate cancer cells were trypsinized and resuspended in TE buffer (10 mM Tris-HCl 7.4, 1 mM EDTA). After incubation on ice for 10 min, the lysate was centrifuged and the supernatant was collected.

**Luminescence response of Ir(III) complexes 1**–**6 towards different forms of DNA**. The G-quadruplex DNA-forming sequences (PS2.M) was annealed in Tris-HCl buffer (20 mM Tris, 100 mM KCl, pH 7.0) and were stored at –20 °C before use. Complexes **1**–**5** (1 µM) was added to 5 µM of ssDNA, ctDNA or PS2.M G-quadruplex DNA in Tris-HCl buffer (20 mM Tris, pH 7.0).

**Table S1** DNA sequences used in this project:

|  | Sequence |
| --- | --- |
| HP1 | 5′-G_2_TACGTG_2_CTGAG_2_C_2_AGTG_3_TAG_3_CG_3_T_2_G_2_C_2_TCAGC_2_ACGTAC_2_-3′ |
| HP2 | 5′-G_2_TACGTG_2_CTGAG_2_C_2_A_2_CGTG_3_TAG_3_CG_3_T_2_G_2_C_2_TCAGC_2_ACGTAC_2_-3′ |
| HP3 | 5′-ACGTG_2_CTGAG_2_C_2_AGTG_3_TAG_3_CG_3_T_2_G_2_C_2_-3′ |
| HP4 | 5′-ACGTG_2_CTGAG_2_C_2_A_2_CGTG_3_TAG_3_CG_3_T_2_G_2_C_2_-3′ |
| HP5 | 5′-ACGTG_2_CTGAG_2_C_2_AGTG_3_TAG_3_CG_3_T_2_G_2_C_2_TCAGC_2_ACGT-3′ |
| PS2.M | 5′-GTG_3_TAG_3_CG_3_T_2_G_2_-3′ |
| CCR5-DEL | 5′-CTCAT_4_C_2_ATACAT_2_A_3_GATAGTCAT-3′ |
| ds26 | 5′-CA_2_TCG_2_ATCGA_2_T_2_CGATC_2_GAT_2_G-3′ |
| ds17 | 5′-C_2_AGT_2_CGTAGTA_2_C_3_-3′  5′-G_3_T_2_ACTACGA_2_CTG_2_-3′ |
| FPS2.MT | 5′-FAM-GTG_3_TAG_3_CG_3_T_2_G_2_-TAMRA-3′ |
| F10T | 5′-FAM-TATAGCTA-HEG-TATAGCTATAT-TAMRA-3′ |
| HP1-M1 | 5′- G_2_TA***ATAC***GCTGAG_2_C_2_AGTG_3_TAG_3_CG_3_T_2_G_2_C_2_TCAGC***GTAT***TAC_2_-3′ |
| HP1-M2 | 5′- G_2_TACGTG_2_CTGAG_2_C_2_AGT***A***_3_TA***A***_3_CG_3_T_2_G_2_C_2_TCAGC_2_ACGTAC_2_-3′ |

The italic bold bases are mutant bases.

**Table S2** Photophysical properties of iridium(III) complexes **1**–**6**.

| Complex | Quantum yield | λ_em_/ nm | Life time/ µs | UV/vis absorption  λ_abs_ / nm (ε/ dm^3^mol^–1^cm^–1^) |
| --- | --- | --- | --- | --- |
| **1** | 0.0633 | 490 | 4.836 | 257 (6.2 × 10^4^), 316 (2.4 × 10^4^),  408 (3.2 × 10^3^) |
| **2** | 0.068 | 525 | 4.284 | 224 (1.5 × 10^5^), 254 (1.25 × 10^5^),  329 (5.0 × 10^4^), 423 (9.2 × 10^3^) |
| **3** | 0.164 | 575 | 2.735 | 260 (1.6 × 10^4^), 284 (2.0 × 10^4^),  340 (1.18 × 10^4^), 447 (3.8 × 10^3^) |
| **4** | – | – | – | 224 (2.64 × 10^4^), 254 (5.48 × 10^4^), 271 (4.68 × 10^4^), 316 (2.32 × 10^4^) |
| **5** | 0.184 | 575 | 4.832 | 260 (2.6 × 10^4^), 282 (3.64 × 10^4^),  346 (1.46 × 10^4^), 445 (4.0× 10^3^) |
| **6** | 0.069 | 490 | 4.597 | 259 (8.4 × 10^4^), 310 (4.0 × 10^4^),  402 (8.0 × 10^3^) |

**Table S3** DNA sequences used in loop effect experiments:

|  | n | Sequence |
| --- | --- | --- |
| 5′-side loop | 1 | 5′-GTG_3_AG_3_CG_3_T_2_G_2_-3′ |
|  | 2 | 5′-GTG_3_TAG_3_CG_3_T_2_G_2_-3′ |
|  | 3 | 5′-GTG_3_TATG_3_CG_3_T_2_G_2_-3′ |
|  | 4 | 5′-GTG_3_T_2_ATG_3_CG_3_T_2_G_2_-3′ |
|  | 5 | 5′-GTG_3_T_2_AT_2_G_3_CG_3_T_2_G_2_-3′ |
|  | 6 | 5′-GTG_3_T_3_AT_2_G_3_CG_3_T_2_G_2_-3′ |
|  | 7 | 5′-GTG_3_T_3_AT_3_G_3_CG_3_T_2_G_2_-3′ |
|  | 9 | 5′-GTG_3_T_4_AT_4_G_3_CG_3_T_2_G_2_-3′ |
|  | 11 | 5′-GTG_3_T_5_AT_5_G_3_CG_3_T_2_G_2_-3′ |
|  | 13 | 5′-GTG_3_T_6_AT_6_G_3_CG_3_T_2_G_2_-3′ |
|  | 15 | 5′-GTG_3_T_7_AT_7_G_3_CG_3_T_2_G_2_-3′ |
|  | 17 | 5′-GTG_3_T_8_AT_8_G_3_CG_3_T_2_G_2_-3′ |
| central loop | 1 | 5′-GTG_3_TAG_3_CG_3_T_2_G_2_-3′ |
|  | 2 | 5′- GTG_3_TAG_3_TCG_3_T_2_G_2_-3′ |
|  | 3 | 5′- GTG_3_TAG_3_TCTG_3_T_2_G_2_-3′ |
|  | 4 | 5′- GTG_3_TAG_3_T_2_CTG_3_T_2_G_2_-3′ |
|  | 5 | 5′- GTG_3_TAG_3_T_2_CT_2_G_3_T_2_G_2_-3′ |
|  | 6 | 5′-GTG_3_TAG_3_T_3_CT_2_G_3_T_2_G_2_-3′ |
|  | 7 | 5′-GTG_3_TAG_3_T_3_CT_3_G_3_T_2_G_2_-3′ |
|  | 9 | 5′-GTG_3_TAG_3_T_4_CT_4_G_3_T_2_G_2_-3′ |
|  | 11 | 5′-GTG_3_TAG_3_T_5_CT_5_G_3_T_2_G_2_-3′ |
|  | 13 | 5′-GTG_3_TAG_3_T_6_CT_6_G_3_T_2_G_2_-3′ |
|  | 15 | 5′-GTG_3_TAG_3_T_7_CT_7_G_3_T_2_G_2_-3′ |
|  | 17 | 5′-GTG_3_TAG_3_T_8_CT_8_G_3_T_2_G_2_-3′ |
| 3′-side loop | 1 | 5′-GTG_3_TAG_3_CG_3_TG_2_-3′ |
|  | 2 | 5′-GTG_3_TAG_3_TCG_3_T_2_G_2_-3′ |
|  | 3 | 5′-GTG_3_TAG_3_TCG_3_T_3_G_2_-3′ |
|  | 4 | 5′-GTG_3_TAG_3_TCG_3_T_4_G_2_-3′ |
|  | 5 | 5′-GTG_3_TAG_3_TCG_3_T_5_G_2_-3′ |
|  | 6 | 5′-GTG_3_TAG_3_TCG_3_T_6_G_2_-3′ |
|  | 7 | 5′-GTG_3_TAG_3_TCG_3_T_7_G_2_-3′ |
|  | 9 | 5′-GTG_3_TAG_3_TCG_3_T_9_G_2_-3′ |
|  | 11 | 5′-GTG_3_TAG_3_TCG_3_T_11_G_2_-3′ |
|  | 13 | 5′-GTG_3_TAG_3_TCG_3_T_13_G_2_-3′ |
|  | 15 | 5′-GTG_3_TAG_3_TCG_3_T_15_G_2_-3′ |
|  | 17 | 5′-GTG_3_TAG_3_TCG_3_T_17_G_2_-3′ |

**Table S4** Comparison of the luminescence-based detections for transcription factors activity assay reported in recent years.

| Transcription factor | Method | Detection limit | Reference |
| --- | --- | --- | --- |
| NF-kB | An Ag+-stabilized self-assembly triplex DNA molecular switch (modified DNA) | 25 pM | [6](#_ENREF_5) |
| NF-kB | A graphene oxide (GO)-based molecular beacon for DNA-binding transcription factor detection | 1 nM | [7](#_ENREF_6) |
| NF-κB | A highly selective, label-free, homogenous luminescent switch-on probe for the detection of nanomolar transcription factor NF-kappaB | 30 nM | [8](#_ENREF_7) |
| NF-kB | Detection of Transcription Factors using transcription-mediated isothermally exponential amplification-induced chemiluminescence | 6.03 fM | [9](#_ENREF_8) |
| TBP,  Myc-Max,  NF-kB | Transcription factor beacons for the quantitative detection of DNA binding activity (modified DNA) | ------- | [10](#_ENREF_9) |
| NF-κB | Detection of transcription factors by isothermal exponential amplification-based colorimetric assay | 3.8 pM | [11](#_ENREF_10) |
| E. coli SSB | A molecular beacon, detection DNA-protein interaction (modified DNA) | 0.2 nM | [12](#_ENREF_11) |
| CAP | Molecular beacons for detecting  DNA binding proteins (modified DNA) | 19.6 nM^a^ | [13](#_ENREF_12) |
| NF-kB | Label-free and enzyme-free detection of  transcription factors with graphene oxide fluorescence switch-based multifunctional G-quadruplex-hairpin probe | 0.2 nM | [14](#_ENREF_13) |

1. The lowest detectable concentration.

**Fig. S1** Luminescence response of complexes **1**–**6** (1 μM) in 10 mM Tris buffer (pH 7.4) in the presence of 5 µM ssDNA (CCR5-DEL), 5 µM ds17 or 5 µM PS2.M G-quadruplex, respectively. PS2.M G-quadruplex was pre-annealed in Tris buffer (10 mM, 100 mM KCl, pH 7.4).


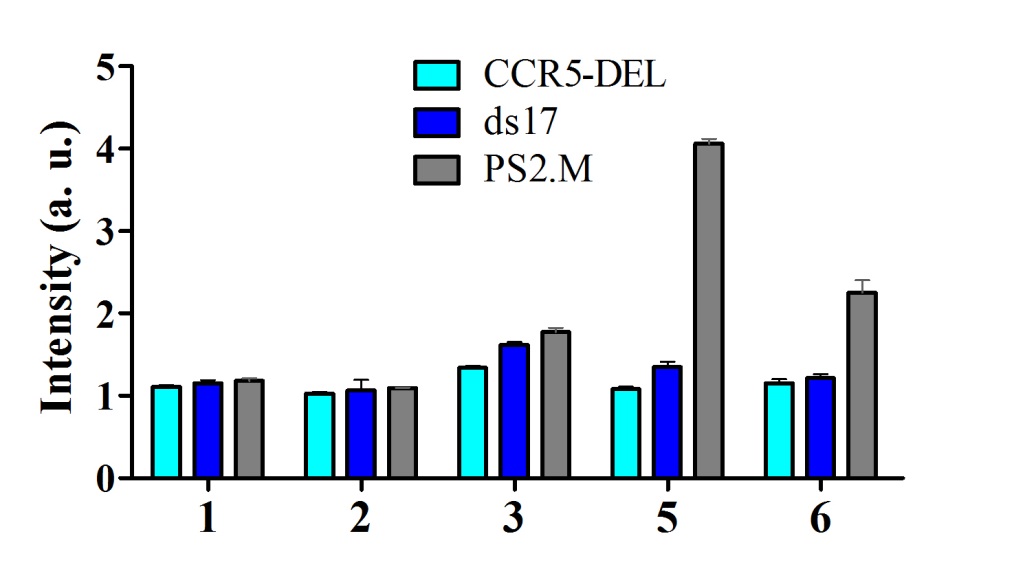


**Fig. S2** UV/Vis absorption of (a) complex **1** and its N^N ligand di(pyridin-2-yl)amine, C^N ligand ppy (b) complex **2** and its N^N ligand di(pyridin-2-yl)amine, C^N ligand baq (c) complex **3** and its N^N ligand di(pyridin-2-yl)amine, C^N ligand phq (d) complex **4** and its N^N ligand di(pyridin-2-yl)amine, C^N ligand ppyr (e) complex **5** and its N^N ligand tpptda, C^N ligand phq (f) complex **6** and its N^N ligand tpptda, C^N ligand ppy. All of the compounds are (2.5 µM) in acetonitrile solution at 298 K.


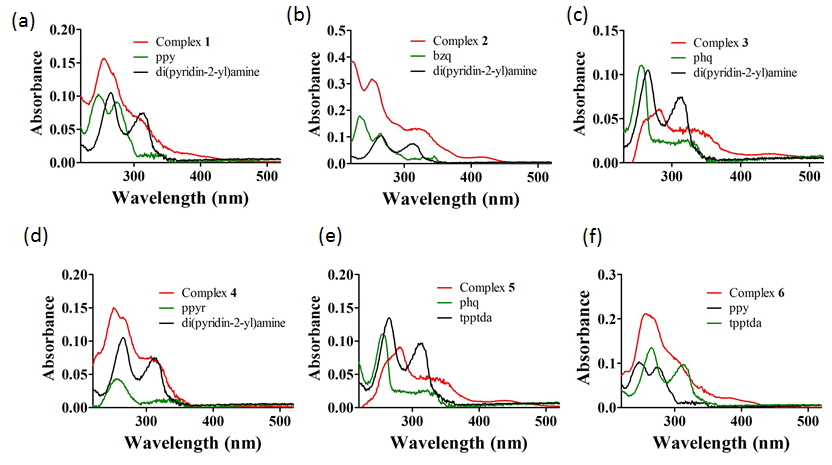


**Scheme S1** Synthetic pathway of dinuclear iridium(III) complexes **5** and **6**.

**
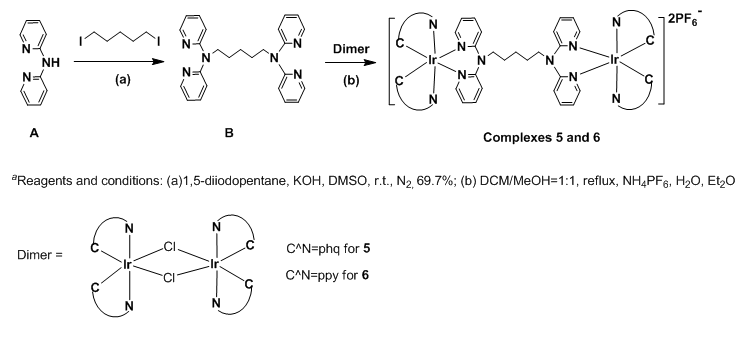
**

**Fig. S3** (a) G4-FID titration trend of DNA duplex ds17 and G-quadruplex DNA PS2.M with the increasing concentration of complex **5** in Tris-HCl buffer. (b) Melting curve of labelled PS2.M G-quadruplex DNA (0.2 μM) in the absence or presence of **5** (3 μM). (c) Melting curve of F10T dsDNA (0.2 μM) in the absence or presence of **5** (3 μM). (d) Melting curve of labelled PS2.M (0.2 μM) in the presence of **5** (3 μM) with addition of ssDNA (2 μM) or ds26 (2 μM).

**
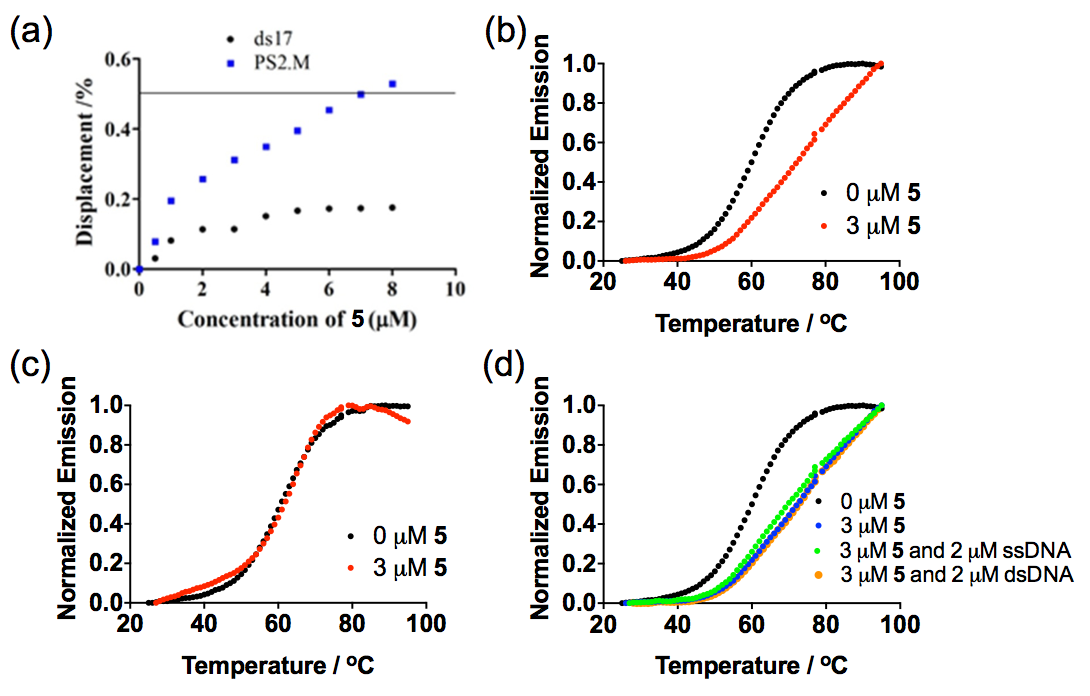
**

**Fig. S4** Luminescence enhancement of complex **5** as a function of loop size (a) 5′-side loop: 5′-GTG_3_**T_x_AT_y_**G_3_CG_3_T_2_G_2_-3′ (n = 1 + x + y = 1–7, 9, 11, 13, 15 and 17) (b) central loop: 5′-GTG_3_TAG_3_**T_x_CT_y_**G_3_T_2_G_2_-3′ (n = 1 + x + y =1–7, 9, 11, 13, 15 and 17) and (c) 3′-side loop 5′- GTG_3_TAG_3_CG_3_**T_n_**G_2_-3′ (n = 1–7, 9, 11, 13, 15 and 17) (in nucleotides, the bold parts are the loop parts of PS2.M. The specific sequences are in Table S3).


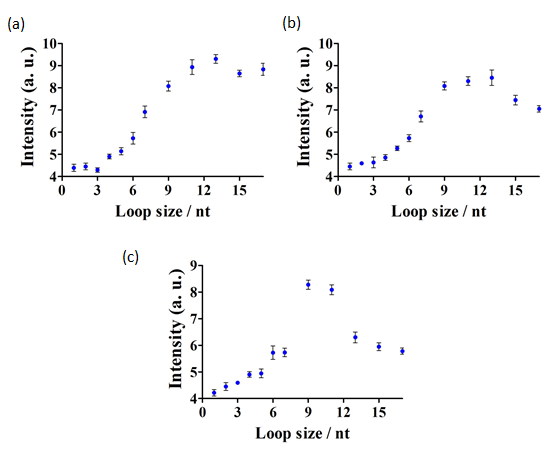


**Fig. S5** The secondary structures used in this project. (a) HP1, (b) HP2, (c) HP3, (d) HP4 and (e) HP5 (The structures are simulated by the software DNAMAN 6.0.3.99, and all the structures were simulated without consideration of ions such as K^+^, Na^+^, Mg^2+^, *et. al*.)


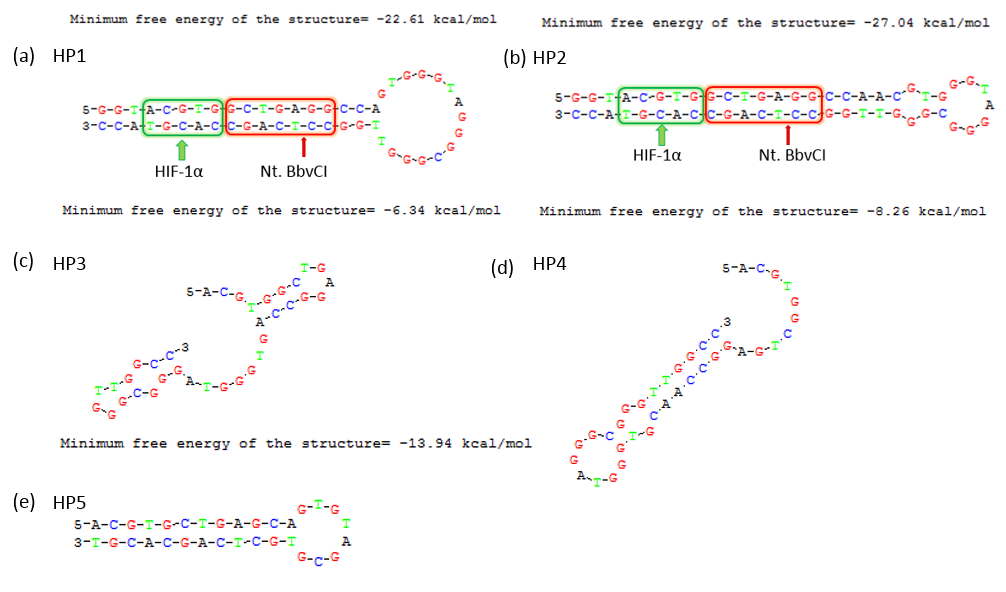


**Fig. S6** Relative luminescence response of the detection system with HIF-1α (80 nM) in the presence of 0.1 µM of hairpin DNA HP1 or 0.1 µM of hairpin DNA HP2, ([complex **5**] = 0.5 µM).

**
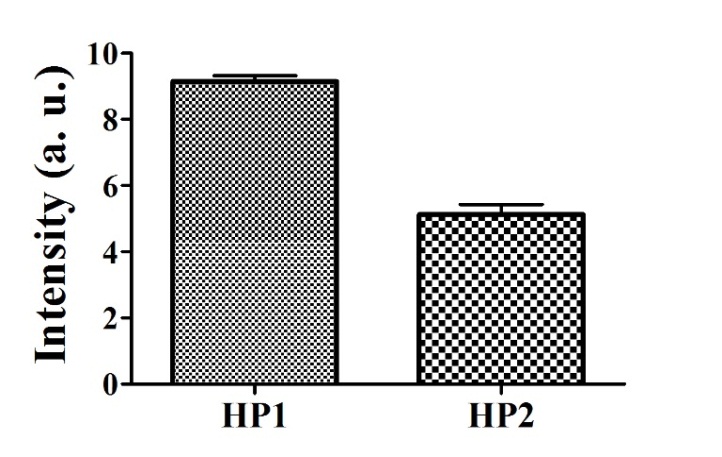
**

**Fig. S7** (a) Luminescence response of the system with the complex alone ([complex **5**] = 0.5 µM) in the absence and presence of HIF-1α (120 nM), (b) Luminescence response of the system with the complex alone ([complex **5**] = 0.5 µM) in the presence of 0.5µM HP1 or in the presence of 0.5 µM HP1 + ExoIII (50 U/mL) + ExoI (40 U/mL).


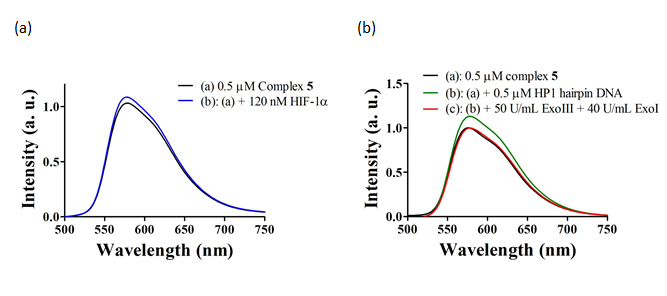


**Fig. S8** Relative luminescence response of complex **5** (1 μM) in the presence of HIF-1α (80 nM) and hairpin DNA HP1 (0.1 μM) or mutant hairpin DNA HP1-M1 (0.1 μM) / HP1-M2 (0.1 μM). Experimental conditions: 0.5 μM of complex **5**, 50 units ExoIII, 40 units ExoI and 25 units Nt, BbvCI in Tris buffer (10 mM, 100 mM KCl, pH 7.4).


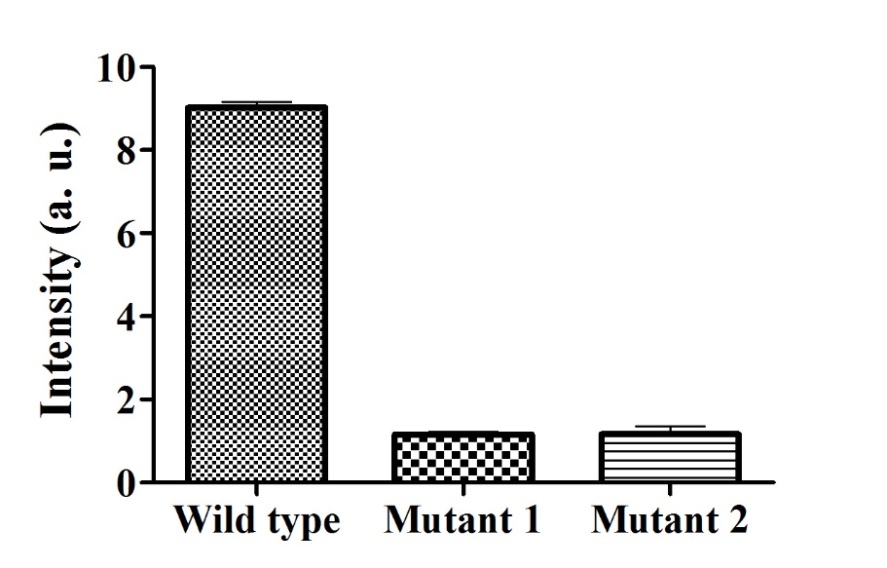


**Fig. S9** Circular dichroism (CD) spectrum of 1 μM of hairpin DNA in the absence (black) or presence (blue) of 300 nM HIF-1α recorded in Tris buffer (10 mM, 100 mM KCl, pH 7.4).


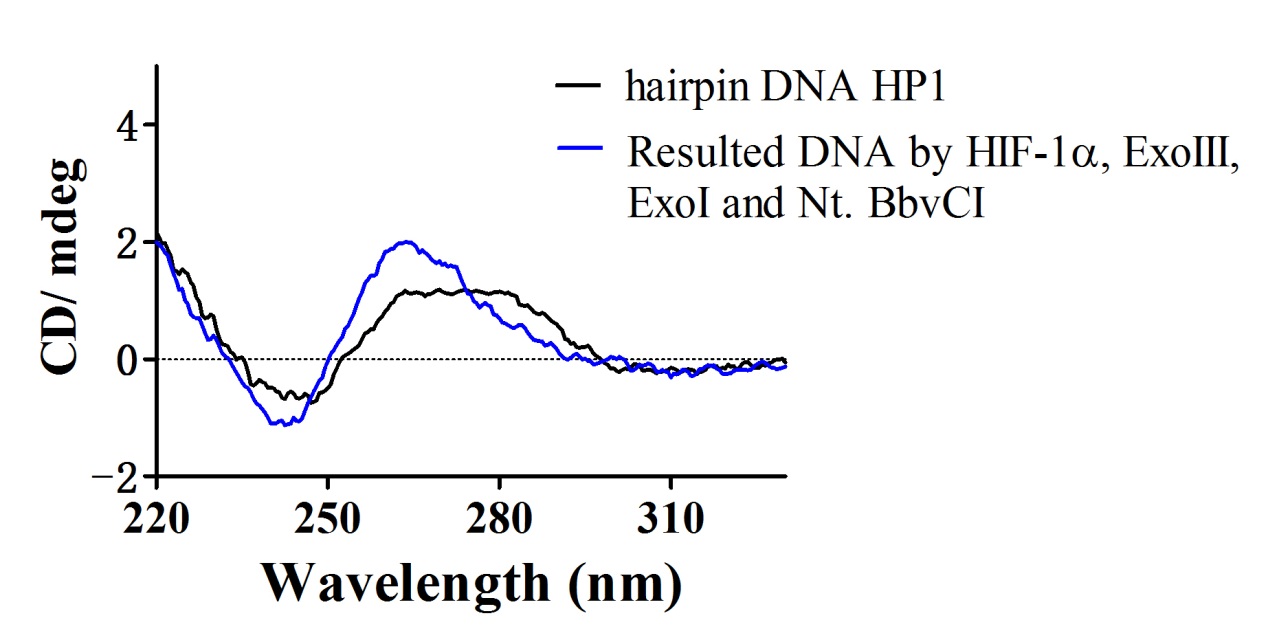


**Fig. S10** Emission spectral traces of complex **5** (0.5 μM) and hairpin DNA (0.10 μM) upon incubation with 5 nM HIF-1α in Tris-HCl buffer (10 mM, 100 mM KCl, pH 7.4), showing a signal-to-noise ratio greater than 3.


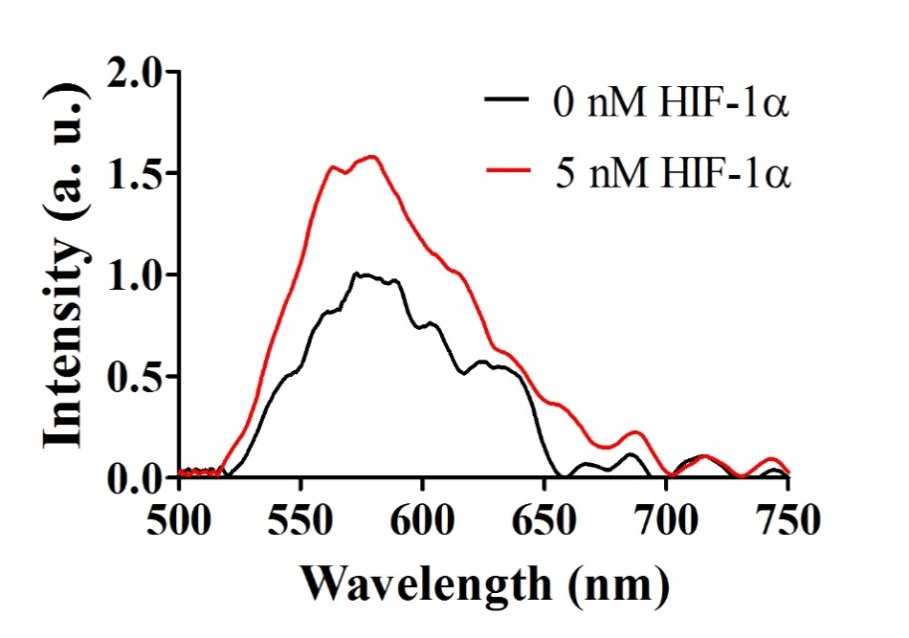


**Fig. S11** Selectivity of the G-quadruplex-based detection platform for HIF-1α over G-quadruplex binding protein (a) HIF-1α (b) thrombin (c) insulin. The concentration of HIF-1α is 80 nM and the concentrations of thrombin and insulin are 0.8 μM.


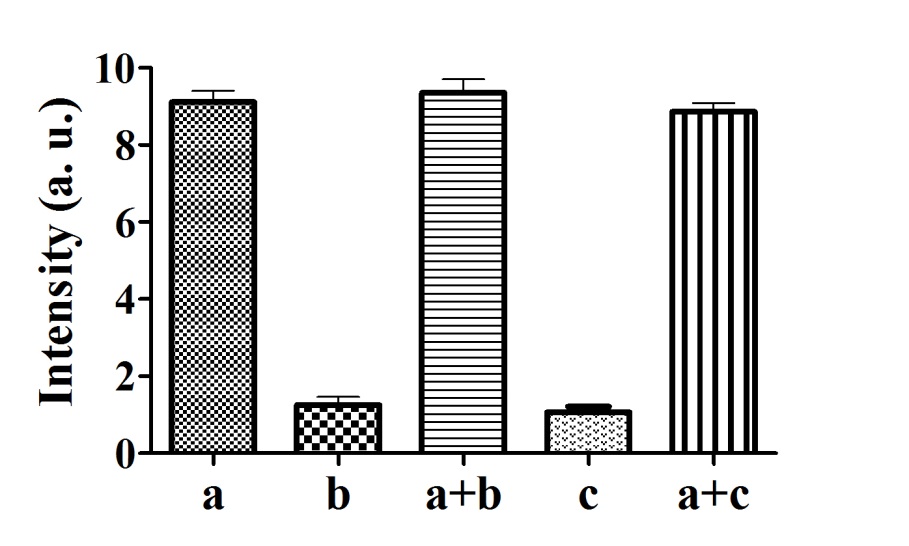


**References**

1. L. Lu, D. S.-H. Chan, D. W. Kwong, H.-Z. He, C.-H. Leung and D.-L. Ma, *Chem. Sci.*, 2014, **5**, 4561-4568.

2. D. Monchaud, C. Allain, H. Bertrand, N. Smargiasso, F. Rosu, V. Gabelica, A. De Cian, J.-L. Mergny and M.-P. Teulade-Fichou, *Biochimie*, 2008, **90**, 1207-1223.

3. P. Yang, A. De Cian, M. P. Teulade‐Fichou, J. L. Mergny and D. Monchaud, *Angew. Chem. Int. Ed.*, 2009, **121**, 2222-2225.

4. Q. Zhao, S. Liu, M. Shi, C. Wang, M. Yu, L. Li, F. Li, T. Yi and C. Huang, *Inorg. Chem.*, 2006, **45**, 6152-6160.

5. Jpn. Kokai Tokkyo Koho, JP 2005298483 A 20051029, 2005.

6. D. Zhu, J. Zhu, Y. Zhu, L. Wang and W. Jiang, *Chem. Commun.*, 2014, **50**, 14987-14990.

7. J.-J. Liu, X.-R. Song, Y.-W. Wang, G.-N. Chen and H.-H. Yang, *Nanoscale*, 2012, **4**, 3655-3659.

8. D.-L. Ma, T. Xu, D. S.-H. Chan, B. Y.-W. Man, W.-F. Fong and C.-H. Leung, *Nucleic Acids Res.*, 2011, **39**, e67-e67.

9. X. Ma, Z. Chen, J. Zhou, W. Weng, O. Zheng, Z. Lin, L. Guo, B. Qiu and G. Chen, *Biosens. Bioelectron.*, 2014, **55**, 412-416.

10. A. Vallée-Bélisle, A. J. Bonham, N. O. Reich, F. Ricci and K. W. Plaxco, *J. Am. Chem. Soc.*, 2011, **133**, 13836-13839.

11. Y. Zhang, J. Hu and C.-y. Zhang, *Anal. Chem.*, 2012, **84**, 9544-9549.

12. J. J. Li, X. Fang, S. M. Schuster and W. Tan, *Angew. Chem. Int. Ed.*, 2000, **39**, 1049-1052.

13. T. Heyduk and E. Heyduk, *Nat. Biotechnol.*, 2002, **20**, 171-176.

14. D. Zhu, L. Wang, X. Xu and W. Jiang, *Biosens. Bioelectron.*, 2016, 75, 155-160.
